# Identification and expression profiling of *GAPDH* family genes involved in response to *Sclerotinia sclerotiorum* infection and phytohormones in *Brassica napus*

**DOI:** 10.3389/fpls.2024.1360024

**Published:** 2024-04-30

**Authors:** Jing Xu, Rongbo Wang, Xiong Zhang, Wei Zhuang, Yang Zhang, Jianxin Lin, Penglin Zhan, Shanhu Chen, Heding Lu, Airong Wang, Changjian Liao

**Affiliations:** ^1^ Institute of Crop Research, Fujian Academy of Agricultural Sciences (Fujian Germplasm Resources Center)/Fujian Province Characteristic Dry Crop Variety Breeding Engineering Technology Research Center, Fuzhou, China; ^2^ State Key Laboratory of Ecological Pest Control for Fujian and Taiwan Crops, Fujian Agriculture and Forestry University, Fuzhou, China; ^3^ Fujian Key Laboratory for Monitoring and Integrated Management of Crop Pests, Institute of Plant Protection, Fujian Academy of Agricultural Sciences, Fuzhou, China; ^4^ The Key Laboratory of Biology and Genetic Improvement of Oil Crops, The Ministry of Agriculture and Rural Affairs of the PRC, Oil Crops Research Institute, Chinese Academy of Agricultural Sciences, Wuhan, China

**Keywords:** *Brassica napus*, *GAPDH*, gene family, *Sclerotinia sclerotiorum*, subcellular localization, nuclear translocation

## Abstract

Glyceraldehyde 3-phosphate dehydrogenase (GAPDH) is a crucial enzyme in glycolysis, an essential metabolic pathway for carbohydrate metabolism across all living organisms. Recent research indicates that phosphorylating GAPDH exhibits various moonlighting functions, contributing to plant growth and development, autophagy, drought tolerance, salt tolerance, and bacterial/viral diseases resistance. However, in rapeseed (*Brassica napus*), the role of GAPDHs in plant immune responses to fungal pathogens remains unexplored. In this study, 28 genes encoding GAPDH proteins were revealed in *B. napus* and classified into three distinct subclasses based on their protein structural and phylogenetic relationships. Whole-genome duplication plays a major role in the evolution of *BnaGAPDHs*. Synteny analyses revealed orthologous relationships, identifying 23, 26, and 26 *BnaGAPDH* genes with counterparts in *Arabidopsis*, *Brassica rapa*, and *Brassica oleracea*, respectively. The promoter regions of 12 *BnaGAPDHs* uncovered a spectrum of responsive elements to biotic and abiotic stresses, indicating their crucial role in plant stress resistance. Transcriptome analysis characterized the expression profiles of different *BnaGAPDH* genes during *Sclerotinia sclerotiorum* infection and hormonal treatment. Notably, *BnaGAPDH17*, *BnaGAPDH20*, *BnaGAPDH21*, and *BnaGAPDH22* exhibited sensitivity to *S. sclerotiorum* infection, oxalic acid, hormone signals. Intriguingly, under standard physiological conditions, *BnaGAPDH17*, *BnaGAPDH20*, and *BnaGAPDH22* are primarily localized in the cytoplasm and plasma membrane, with *BnaGAPDH21* also detectable in the nucleus. Furthermore, the nuclear translocation of *BnaGAPDH20* was observed under H_2_O_2_ treatment and *S. sclerotiorum* infection. These findings might provide a theoretical foundation for elucidating the functions of phosphorylating GAPDH.

## 1 Introduction

Glycolysis is a crucial metabolic process involved in carbohydrate metabolism ubiquitous across all living organisms (Danshina et al., 2001). Glyceraldehyde 3-phosphate dehydrogenase (GAPDH) plays a central role in this pathway, facilitating the NAD-dependent conversion of glyceraldehyde 3-phosphate (G3P) to 1,3-bisphosphoglyceric acid (Bruns and Gerald, 1976). Animal cells have only one isoform of GAPDH (Nicholls et al., 2012; Zhang et al., 2020), but plants exhibit multiple isoforms of GAPDHs, each encoded by distinct genes and residing in specific subcellular compartments (Zaffagnini et al., 2013). For instance, the *Arabidopsis thaliana* genome encodes eight GAPDHs, including seven phosphorylating GAPDHs and one nonphosphorylating GAPDH. The phosphorylating GAPDHs consist of chloroplast photosynthetic GAPDH (GAPA1, GAPA2, and GAPB), cytosolic glycolytic GAPDHs (GAPC1 and GAPC2), and plastidic glycolytic GAPDHs (GAPCp1 and GAPCp2). The nonphosphorylating GAPDH is referred to as NADP-dependent nonphosphorylating cytosolic GAPDH (NP-GAPDH) (Zaffagnini et al., 2013). Comprehensive studies have led to the identification and functional elucidation of various *GAPDH* gene families across numerous plant species, including *Arabidopsis* (Rius et al., 2006; Holtgrefe et al., 2008; Muñoz-Bertomeu et al., 2009, 2010; Muñoz-Bertomeu et al., 2011; Guo et al., 2012; Kim et al., 2020), rice (Zhang et al., 2011), wheat (Zeng et al., 2016; Ying et al., 2017), tobacco (Han et al., 2015), potato (Liu et al., 2017), sweet orange (Miao et al., 2019), strawberry (Luo et al., 2020), and cassava (Zeng et al., 2018).

*GAPDHs* have traditionally served as internal reference genes (Morgante et al., 2011; Kozera and Rapacz, 2013; Zhu et al., 2014). Nonetheless, recent findings indicate that the expression levels of *GAPDHs* vary under different stress scenarios (Bai et al., 2012), a revealing numerous moonlighting functions including membrane trafficking (Tisdale, 2001), mRNA stability (Zeng et al., 2014), DNA repair (Demarse et al., 2009), transcriptional expression (Zheng et al., 2003) signal transduction (Harada et al., 2007), cellular apoptosis (Hara et al., 2005),membrane fusion (Nakagawa et al., 2002), drought stress (Zhang et al., 2020), autophagy (Wang et al., 2022), heat stress (Kim et al., 2020) and immunity (Henry et al., 2015). For example, in *Arabidopsis*, *AtGAPDHs*, including *AtGAPA*, *AtGAPCs*, and *AtGAPCps*, regulate the accumulation of reactive oxygen species (ROS) and cell death when exposed to the bacterial pathogen *Pseudomonas syringae*, thus negatively regulating disease resistance (Henry et al., 2015). Moreover, *NbGAPCs* in tobacco are known for their multifunctional roles in regulating autophagy, hypersensitive response, and plant innate immunity (Han et al., 2015). On the contrary, *MeGAPCs* in cassava play a contrasting role by negatively regulating plant disease resistance against *Xanthomonas axonopodis* pv. *manihotis* (Xam) by interacting with MeATG8b and MeATG8e (Zeng et al., 2018). Furthermore, cytosolic *GAPDHs* are also involved in viral infection (Kaido et al., 2014).

One mechanism underlying GAPDHs’ action in stress response is their stress-induced nuclear translocation (Sirover, 2021). In *Arabidopsis*, a portion of cytosolic GAPDHs accumulates in the nucleus in response to various stimuli, including cadmium, bacterial flagellin, phosphatidic acid, and hydrogen sulfide (Vescovi et al., 2013; Henry et al., 2015; Aroca et al., 2017). This nuclear accumulation of cytosolic GAPDHs was also noted in tobacco BY-2 (bright-yellow 2) cells subjected to programmed cell death (PCD) triggers, such as long-chain bases (Testard et al., 2016). Given that GAPC lacks a nuclear localization signal, the post-translational modifications of specific amino acid residues are believed to be crucial for its intracellular translocation. In mammals, when cells are continuously exposed to a stressor, an increase in the level of nitrosative stress beyond a certain threshold leads to the nitrosylation of the catalytic Cys150 of rat GAPDH (forming SNO-GAPDH) (Hara et al., 2005). SNO-GAPDH is recognized by Siah1 which contains a nuclear localization signal and mediates the translocation of the SNO-GAPDH-Siah1 complex to the nucleus (Hara et al., 2005). It is reported that the cytosolic GAPDHs AtGAPC1 and AtGAPC2 undergo redox-dependent cysteine modifications, leading to the enzymes’ inactivation in glycolysis (Holtgrefe et al., 2008). Additionally, GAPDH is thought to function as an H_2_O_2_ sensor, initiating the protective oxidative stress response and helping reestablish cellular homeostasis. Furthermore, the nuclear presence of cytosolic GAPDH increases under oxidizing conditions in *Arabidopsis* (Schneider et al., 2018). In summary, GAPDH may detect H_2_O_2_ and undergo post-translational modifications, leading to its nuclear translocation, possibly in conjunction with its moonlighting functions.

*Brassica napus* is one of the most important oil crops in China and one of the four major oil crops globally. It belongs to the Brassicaceae family and is an allotetraploid (2*n* = 38, AACC) resulting from hybridization between *Brassica rapa* (2*n* = 20, AA) and *Brassica oleracea* (2*n* = 18, CC) around 7500 years ago (Bayer et al., 2017; Ding et al., 2021). The *B. napus* genome has evolved through ancient polyploidization events, recent hybridization, and gene loss, resulting in approximately 100,000 genes (De Grassi et al., 2008). This evolutionary history and the close relationship of *B. napus* with *A. thaliana* make *B. napus* an ideal model for studying gene family evolution. *B. napus* faces constant challenges by various pathogenic microorganisms, including *Sclerotinia sclerotiorum* (de Bary.), which causes severe stem rot (SSR). This disease leads to yield losses of 10%–20% and can reach up to 80% in some seasons (Yang, 1959; Li et al., 2006; Mei et al., 2011). Although measures like fungicidal control and crop rotation can mitigate this issue, they are often insufficient due to the long survival of sclerotia in the soil and the wide host range of *S. sclerotiorum*. Consequently, breeding new resistant varieties is essential for managing SSR in the future (Ding et al., 2021). Understanding the interaction mechanism between *B. napus* and *S. sclerotiorum* is fundamental to developing disease-resistant varieties.

In all kingdoms of life, stress situations caused by pathogens, such as *S. sclerotiorum*, have been linked to increased cellular levels of ROS (Hildebrandt et al., 2015). GAPDHs are likely involved in this process in *B. napus*. However, comprehensive information about the *GAPDH* gene family in this crop species is lacking. Therefore, in this study, an *in silico* approach was first used to identify and characterize the phosphorylating *GAPDH* family in *B. napus* and then systematically analyze their expression patterns under various stress conditions, including *S. sclerotiorum* infection and treatment with different hormones and oxalic acid (OA). Specifically, four genes highly induced by these stimuli were selected to observe their subcellular localization and nuclear translocation under normal and stress conditions. In this study, *B. napus* was used as the research material to clarify the quantity, nature, general relationship, and function of phosphorylating GAPDH proteins in *B. napus*. This research provides a theoretical foundation for clarifying the functions of phosphorylating *GAPDHs.*

## 2 Materials and methods

### 2.1 Identification of *GAPDH* family genes

Protein sequences from *B. napus*, *B. rapa*, and *B. oleracea* were used to identify *GAPDH* genes. Initially, the *B. napus* v5.0 protein set was downloaded from http://www.genoscope.cns.fr/brassicanapus, *B. rapa* “Z1” and *B. oleracea* “HDEM” protein sets were downloaded from https://www.genoscope.cns.fr/externe/plants/chromosomes.html. Next, the six GAPDH protein sequences of *A. thaliana* downloaded from TAIR (https://www.*Arabidopsis*.org/) were used as queries to perform BLASTp searches against the protein sequences of each species, employing an E-value cutoff of 1e-10. Subsequently, HMMER v3.0 was employed for an HMM search against the local protein database, using the specific Gp_dh_N domain (PF00044) and Gp_dh_C domain (PF02800) HMM profiles obtained from the Pfam database (http://pfam.xfam.org/search), with the default parameters and an E-value cutoff of 1e−5. Subsequently, all putative GAPDHs were validated by batch-CD search (http://www.ncbi.nlm.nih.gov/Structure/cdd/wrpsb.cgi), Pfam (http://pfam.xfam.org/), and SMART (http://smart.embl-heidelberg.de/) databases. Furthermore, the biochemical parameters of BnaGAPDHs were determined using the ProtParam tool (https://web.expasy.org/protparam/, accessed on August 11, 2021). Finally, the subcellular localizations of BnaGAPDHs were predicted using the Plant-mPLoc tool, as described by KuoChen and HongBin in 2010.

### 2.2 Multiple sequence alignments and phylogenetic analysis

Alignment of the full-length GAPDH protein sequences from *B. napus*, *B. rapa*, *B. oleracea*, and *A. thaliana* was performed using the MAFFT online server (https://www.ebi.ac.uk/Tools/msa/mafft/) (Madeira et al., 2019; Rozewicki et al., 2019). A maximum-likelihood phylogenetic tree was constructed using IQ-TREE (Nguyen et al., 2015). For tree construction, the best-fit model, JTT+G4, was chosen based on the Bayesian Information Criterion with ModelFinder (integrated within IQ-TREE) (Kalyaanamoorthy et al., 2017). Both the Ultrafast Bootstrap and the Shimodaira-Hasegawa approximate likelihood ratio test (SH-aLRT) were conducted with 1000 replicates. The resulting tree file was visualized using FigTree V1.4.4 (https://github.com/rambaut/figtree/releases).

### 2.3 Promoter sequence, gene structure, and conserved motif analysis

The upstream 2000-bp sequences relative to the start codon of each *BnaGAPDH* gene were obtained to analyze the promoter regions, and the cis-elements within these regions were predicted using the PlantCARE web tool (http://bioinformatics.psb.ugent.be/webtools/plantcare/html/), accessed on 27 July 2021. The findings were visualized using TBtools (V 1.09854).

The exon-intron structures of the *BnaGAPDH* genes were illustrated by the Gene Structure Display Server (GSDS; http://gsds.cbi.pku.edu.cn), following the genome annotation. The conserved motifs within these proteins were identified using the MEME suite (Bailey et al., 2009) (http://meme-suite.org/tools/meme), employing the following parameters: any number of repetitions, optimal motif widths between 6 and 50 residues, and a maximum of 10 motifs. A comprehensive schematic diagram depicting the amino acid motifs and gene structure for each *GAPDH* gene was subsequently assembled using the Advanced Gene Structure View module in TBtools.

### 2.4 Chromosomal distribution, gene duplication, and collinear analysis

The chromosomal distribution of *BnaGAPDHs* was obtained from the gff3 genome annotation file of *B. napus* and visualized using the module Gene Location Visualize from GTF/GFF in TBtool. A tandem duplication case is defined as a homologous gene pair located within a 200-kb region of the same chromosome as well as a separation gap of 20 or fewer genes (Malik et al., 2020). The interspecific and intraspecific collinear analyses were performed with MCScanX (E-value 1e-10) (Wang et al., 2012) and visualized using Multiple Synteny Plot in TBtools (V 1.09854). Nonsynonymous (*Ka*) and synonymous (*Ks*) substitution sites of each duplicated gene pair were calculated using the Ka/Ks calculator in TBtools (V 1.09854).

### 2.5 Transcriptional profile of *BnaGAPDHs* in different tissues and during *S. sclerotiorum* infection

The sclerotia of the fungus *S. sclerotiorum* 1980 were germinated to produce hyphal inoculum on potato dextrose agar (PDA) medium. Sensitive cultivar 84039 and moderately resistant cultivar ZhongShuang9 were grown in pots containing soil and vermiculite (3:1, *v*/*v*) under greenhouse conditions at 23–25°C with a 16/8-h light/dark photoperiod and fertilization with commercial N: K: P (1:1:1) every 10 days. After 4 weeks, three fourths of the leaves were inoculated with agar plugs excised from the edges of growing *S. sclerotiorum* colonies. The samples were collected at 12 and 22 hpi and then sent to Novogene Co., Ltd. (Beijing, China) for RNA extraction, library construction, and transcriptome sequencing on the Illumina sequencing platform. After removing the 5’ and 3′ -adapters, *N >*10% sequences, and low-quality sequences (sequence quality values ≤Q20), the clean data were aligned to the *B. napus* reference genome (https://www.genoscope.cns.fr/brassicanapus/) using TopHat2 (Kim et al., 2013)(http://ccb.jhu.edu/software/tophat/index.shtml). The transcript abundance [fragments per kilobase million (FPKM) value] of each gene was calculated using HTSeq (Anders et al., 2015) (https://htseq.readthedocs.io/en/release_0.11.1/).

*B. napus* cultivar ZhongYou 821 (ZY821) and Westar were generally used as resistant and susceptible doubled haploid lines in response to *S. sclerotiorum* inoculation. The expression data of *BnaGAPDHs* during the inoculation of *S. sclerotiorum* in *B. napus* cultivar ZY821 and cultivar Westar were downloaded from the NCBI GEO database (Accession number GSE81545). Similarly, the expression profile of *BnaGAPDHs* in different tissues/organs of *B. napus* cultivar “Zhongshuang 11” were downloaded from the *B. napus* transcriptome information resource (http://yanglab.hzau.edu.cn/BnTIR/expression_show). The FPKM values of all *BnaGAPDHs* were extracted and submitted to TBtools to generate heatmaps. All of the heatmaps were normalized using log2(value+1).

### 2.6 Plant cultivation, treatments, RNA isolation, and qRT-PCR

The third and fourth leaves of 4-week-old Zhongshuang9 were sprayed with methyl jasmonic acid (MeJA), salicylic acid (SA), and OA or inoculated with *S. sclerotiorum* strain 1980, collected at 0, 6, 12, 24, 36 and 48 hours post-inoculation (hpi), immediately frozen in liquid nitrogen, and stored at −80°C.

The total RNA isolation and purification of samples were performed using an RNAprep Pure Plant Plus Kit (rich in polysaccharides and polyphenolics) (Tiangen, Beijing, China). The RNA isolation for gene expression was done in triplicate for each sample analyzed. RNA integrity was visualized by 1% agarose gel electrophoresis. The concentration and purity of RNAs (OD_260_/OD_280_ ratio > 1.95) were determined with a NanoDrop One microvolume UV-vis spectrophotometer (NanoDrop Technologies, DE, USA). Further, 1 μg of total RNA was reverse transcribed in a 20-μL reaction volume using a PrimeScript RT reagent kit with a gDNA eraser (Takara, Beijing, China) following the manufacturer’s instructions to remove traces of contaminant DNA and prepare cDNA.

Quantitative real-time polymerase chain reaction (qRT-PCR) analysis was used to analyze the expression level of the identified *BnaGAPDHs*. The standard qRT-PCR with SYBR Premix Ex Taq II (TaKaRa, Beijing, China) was repeated at least three times on a CFX96 real-time System (BioRad, Beijing). Primer Premier 6.0 software were used to designed the specific primers of *BnaGAPDH* genes according to their gene sequences, listed in **Supplementary Table S1**. Results were analyzed by the 2^−(ΔΔCt)^ method using the *BnaACTIN* (*BnaC05g34300D*) as the endogenous reference gene (He et al., 2019).

### 2.7 Subcellular localization

The full-length coding sequences of the selected *BnaGAPDH* genes were isolated and linked into the pEarlygate104 vector containing YFP reporter (saved in our laboratory). The competent cells of *Escherichia coli* (DH5α) and *Agrobacterium* (GV3101) were used for the transformation of recombinants. Primers used for gene cloning and vector construction are shown in **Supplementary Table S1**. Agrobacterium-mediated transient expression in tobacco (*Nicotiana benthamiana*) leaves was performed as previously described (Chen et al., 2019). Tobacco leaves injected with *Agrobacterium agrobacterium* for 40-48 h containing recombinant vector were cut into 3 mm^2^ small leaf discs. 4’,6-diamidino-2-phenylindole (DAPI) dyeing solution with 0.1%TritonX-100 mixed in advance was added (CatNo.C1006, Beyotime Biotechnology Co., LTD.) two hours before observing using the laser scanning confocal microscopy (Olympus FV3000, Tokyo, Japan). For treatment of H_2_O_2_, the small leaf discs treated with DAPI were transferred into solution containing10 mM H_2_O_2_ 40 minis before imaging. For inoculation treatment, 36-40 hours after injection of *Agrobacterium*, tobacco leaves were cut and placed in a tray with wetting filter paper back side up. After inoculating the hyphal plugs on the leaves, the plates were covered with a transparent plastic lid and incubated at 22°C for 8-12 hours. Blue fluorescence at the nuclear could be observed under an excitation wavelength of 340nm. The YFP fluorescence was observed under an excitation wavelength of 488 nm.

## 3 Results

### 3.1 Genome-wide identification of *GAPDH* family genes in *Brassica napus*

A BLASTP method using six known Arabidopsis homologs as queries and HMM searches with the Gp_dh_N (PF00044) and Gp_dh_C (PF02800) domains were performed to search the local protein database of B. napus so as to accurately identify *GAPDH* genes. After removing redundant sequences and domain verification, 28 full-length *GAPDH* homologous sequences were identified in the genomes of *B. napus*. Similarly, 14 and 14 full-length *GAPDH* homologous sequences were identified in the genomes of *B. rapa* and *B. oleracea*, respectively.

**Figure 1:**
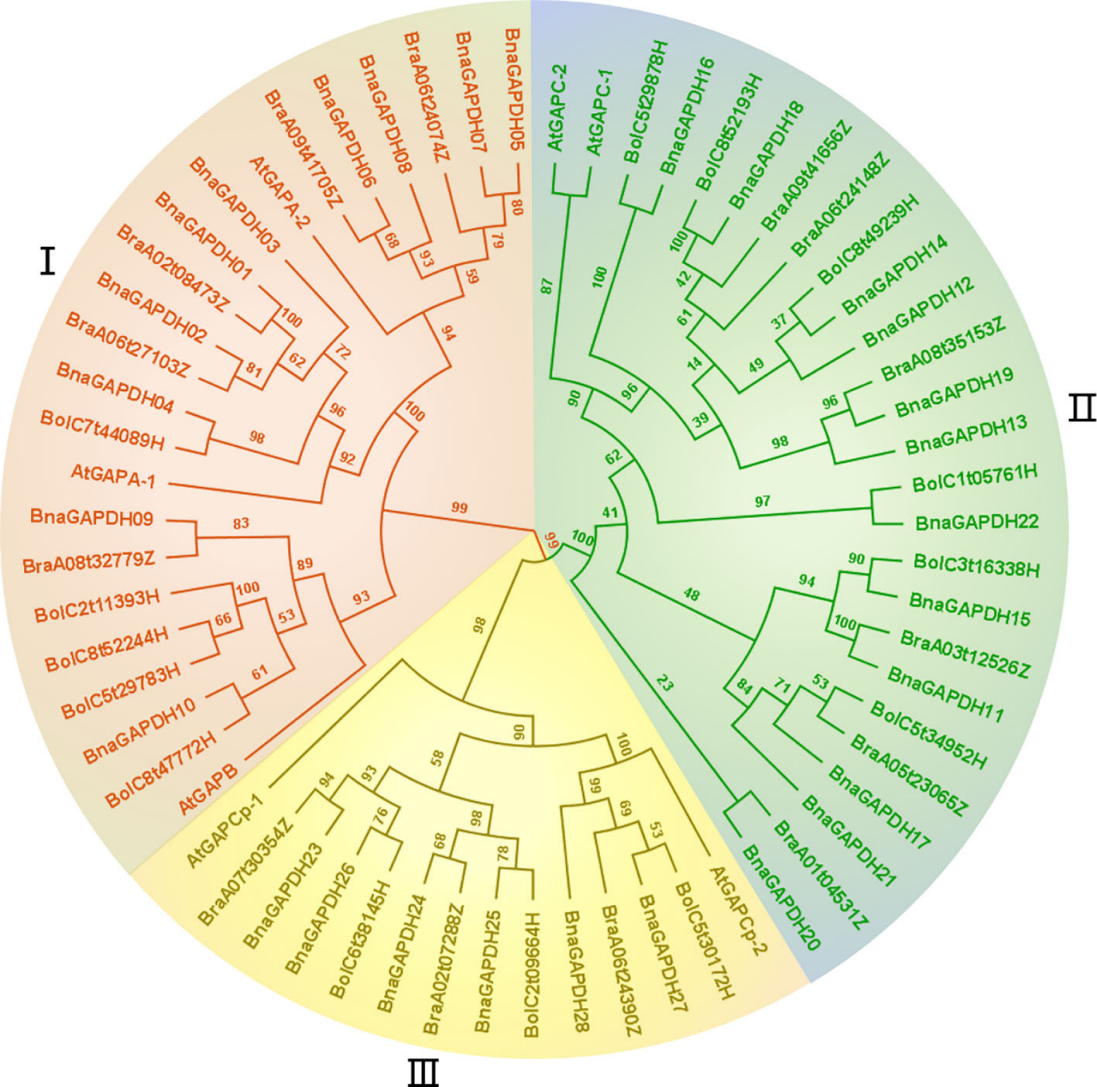
Phylogenetic relationship analysis of *GAPDH* family. The construction of phylogenetic tress using the GAPDH amino acid sequences among *B. napus*, *B. rapa, B. oleracea*, and *A.thaliana*. Phylogenetic tree topology was generated by IQ-Tree online server. The bootstrap value is 1000. All *GAPDH* genes in the phylogenetic tree are colored group-specific.

Like Arabidopsis, 28 *GAPDHs* in *B. napus* were classified into 3 groups based on the domain organization: Group I (GAPA/B), Group II (GAPCs), and Group III (GAPCps) (**Table 1** and **Figure 1**). New names were assigned to 28 *GAPDH* genes in *B. napus* using the prefix “Bna,” followed by “GAPDH” and numbers based on their isoforms and chromosome positions (**Table 1**). As shown in **Table 1**, Group I had 10 members (*BnaGAPDH01* to *BnaGAPDH10*), Group II had 12 members (*BnaGAPDH11* to *BnaGAPDH22*), and Group III had 6 members (*BnaGAPDH23* to *BnaGAPDH28*).

**Table 1:** The characteristics of SNARE genes from *B. napus*.

Group	Gene ID	Gene Name	Gene Features						Protein Features				
			At.Orth^a^	Chr No.^b^	From	To	Exons No.	MW (KDa)	pI	Sub. Loc.^c^	GRAVY	Instability index	Aliphatic index
Group I	BnaA02g28150D	BnaGAPDH01	GAPA1	chrA02	20765457	20767169	6	39.65	8.93	Chl.	-0.038	27.23	93.51
	BnaA06g32740D	BnaGAPDH02	GAPA1	chrA06	21738527	21740415	5	40.89	6.46	Chl.	-0.019	24.87	92.84
	BnaC02g36240D	BnaGAPDH03	GAPA1	chrC02	39344488	39346957	6	40.89	6.46	Chl.	-0.03	26.53	92.08
	BnaC07g23750D	BnaGAPDH04	GAPA1	chrC07	30218651	30220458	5	40.90	6.46	Chl.t	-0.026	25.4	92.08
	BnaA06g07790D	BnaGAPDH05	GAPA2	chrA06	4161459	4163308	5	42.71	7.62	Chl.	-0.046	21.55	91.85
	BnaA09g46780D	BnaGAPDH06	GAPA2	chrA09	31682490	31684580	5	42.60	7.59	Chl.	-0.034	22.18	91.38
	BnaC05g09210D	BnaGAPDH07	GAPA2	chrC05	4979353	4981199	5	42.71	7.62	Chl.	-0.046	21.55	91.85
	BnaC08g40850D	BnaGAPDH08	GAPA2	chrC08	35863233	35865234	5	42.58	7.00	Chl.	-0.026	22.85	91.38
	BnaA08g04610D	BnaGAPDH09	GAPB	chrA08	4361919	4364683	9	47.55	6.76	Chl.	0.001	25.55	93.79
	BnaC08g46180D	BnaGAPDH10	GAPB	chrC08_r	194051	196533	8	42.63	5.59	Chl.	-0.012	21.62	94.08
Group II	BnaA03g28480D	BnaGAPDH11	GAPC1	chrA03	13905612	13907758	8	38.40	7.02	Cyt.	0.002	23.32	91.59
	BnaA06g08510D	BnaGAPDH12	GAPC1	chrA06	4603324	4605705	11	35.29	6.02	Cyt.	-0.163	24.21	90.06
	BnaA08g24270D	BnaGAPDH13	GAPC1	chrA08	17019972	17024175	11	41.08	7.15	Cyt.	-0.158	28.38	89.36
	BnaA09g46260D	BnaGAPDH14	GAPC1	chrA09	31472035	31474433	11	37.06	5.94	Cyt.	-0.161	22.76	89.82
	BnaC03g33610D	BnaGAPDH15	GAPC1	chrC03	20464104	20466211	8	37.87	6.41	Cyt.	-0.064	22.4	90.4
	BnaC05g09880D	BnaGAPDH16	GAPC1	chrC05	5502940	5507477	10	43.22	7.12	Cyt.	-0.052	26.44	94.2
	BnaC05g47450D	BnaGAPDH17	GAPC1	chrC05	42417313	42420056	9	38.42	7.09	Cyt.	-0.128	23.39	86.37
	BnaC08g40330D	BnaGAPDH18	GAPC1	chrC08	35595880	35598379	10	47.55	6.65	Cyt.	-0.083	28.31	90.42
	BnaC08g15900D	BnaGAPDH19	GAPC1	chrC08	20198745	20201398	11	40.77	7.12	Cyt.	-0.089	26.37	91.16
	BnaA01g33660D	BnaGAPDH20	GAPC2	chrA01	22777482	22780291	11	37.84	6.76	Cyt.	-0.108	20.87	91.96
	BnaA05g33200D	BnaGAPDH21	GAPC2	chrA05	22569053	22571493	8	36.60	6.44	Cyt.	-0.118	21	88.69
	BnaC01g40210D	BnaGAPDH22	GAPC2	chrC01	38516889	38519565	11	41.74	7.70	Cyt.	-0.076	22.87	97.15
Group III	BnaA07g20300D	BnaGAPDH23	GAPCP1	chrA07	15952255	15955126	14	44.36	8.93	Cyt.Mit.	-0.05	34.17	83.42
	BnaC01g30110D	BnaGAPDH24	GAPCP1	chrC01	28562498	28565570	14	43.89	8.83	Cyt.Mit.	-0.051	33	85.33
	BnaC02g25850D	BnaGAPDH25	GAPCP1	chrC02	23128343	23131403	14	44.38	8.83	Cyt.Mit.	-0.071	31.48	84.03
	BnaC06g19790D	BnaGAPDH26	GAPCP1	chrC06	21938130	21941473	14	44.48	8.80	Cyt.Mit.	-0.071	35.67	82.26
	BnaA06g10900D	BnaGAPDH27	GAPCP2	chrA06	5690356	5693656	14	45.45	8.64	Cyt.Mit.	-0.096	37.99	82.42
	BnaC05g12400D	BnaGAPDH28	GAPCP2	chrC05	7171844	7175139	14	45.65	7.66	Cyt.Mit.	-0.092	36.7	82.65

^a^ identical to the Arabidopsis homologues at the protein level; ^b^ r represents random; ^c^ subcellular localization predicted using the Plant-mPLoc tool. pI: Isoelectric point; Mw: molecular weight; Len: Length. The colors indicate different groups corresponding with that in **Figure 1**.

Gene characteristics, such as amino acid length, isoelectric point (p*I*), molecular weight (MW), grand average of hydropathy (GRAVY) values, Instability index, aliphatic indexand, and subcellular localization were analyzed (**Table 1**). Among the 28 BnaGAPDHs, BnaGAPDH12 was identified to be the smallest protein with 318 amino acids (aa), whereas the largest one was BnaGAPDH09 (448 aa). The predicted p*I* ranged from 5.59 (BnaGAPDH10) to 8.93 (BnaGAPDH1 and BnaGAPDH23), and the MW ranged from 35.29 kDa (BnaGAPDH12) to 47.55 kDa (BnaGAPDH9 and BnaGAPDH18). The GRAVY score, instability index,and aliphatic index were conserved among the BnaGAPDHs. The GRAVY values reflected that almost all BnaGAPDHs predicted to be hydrophilic proteins (GRAVY values <0). Their instability index were lower than 40, indictcating their stable chracteristics.Relatively, the GAPCP-type proteins were predicted to possesses lower stablity than the other two types of BnaGAPDHs according to their instability indes and aliphatic index scores. The Plant-mPLoc predicted that all of the GAPA/B-type BnaGAPDHs were localized in chloroplasts, while all of the GAPC-types in the cytoplasm.The GAPCp-types may appear in the cytoplasm and mitochondria.

### 3.2 Phylogenetic tree of the *GAPDH* gene family in *B. napus*

A maximum-likelihood phylogenetic tree was generated based on the full-length protein sequences for 60 GAPDHs, including 28 *B. napus*, 14 *B. rapa*, 14 *B. oleracea*, and 7 A*. thaliana* members to further characterize and explore the evolutionary relationships of BnaGAPDHs. According to the classification of AtGAPDHs and the topology of the phylogenetic tree, as described earlier, 60 GAPDH*s* were assigned to 3 groups (I, II, and III), containing 23, 26, and 14 members (**Figure 1**), respectively. Each group contained the corresponding subgroup members from *B. napus*, AtGAPDHs, *B. rapa*, and *B. oleracea*. For example, Group III included AtGAPCp-1; AtGAPCp-2; BnaGAPDH23 to BnaGAPDH28; BraA07t30354Z, BraA02t07288Z, and BraA06t24390Z; BolC6t38145H, BolC2t09664H; and BolC5t30172H. Notably, the number of BnaGAPDHs in each group was equal to the sum of that in *B. rapa* and *B. oleracea*. Also, the BnaGAPDHs had a closer phylogenetic relationship with the BraGAPDHs and BloGAPDHs than AtGAPDHs. These results indicated the existence of three ancestral GAPDH paralogs in the most recent common ancestor of Brassicaceae; these paralogs might have undergone subsequent functional divergence.

### 3.3 Chromosomal distribution, gene duplication, and synteny analysis of *B. napus GAPDH* genes

**Figure 2:**
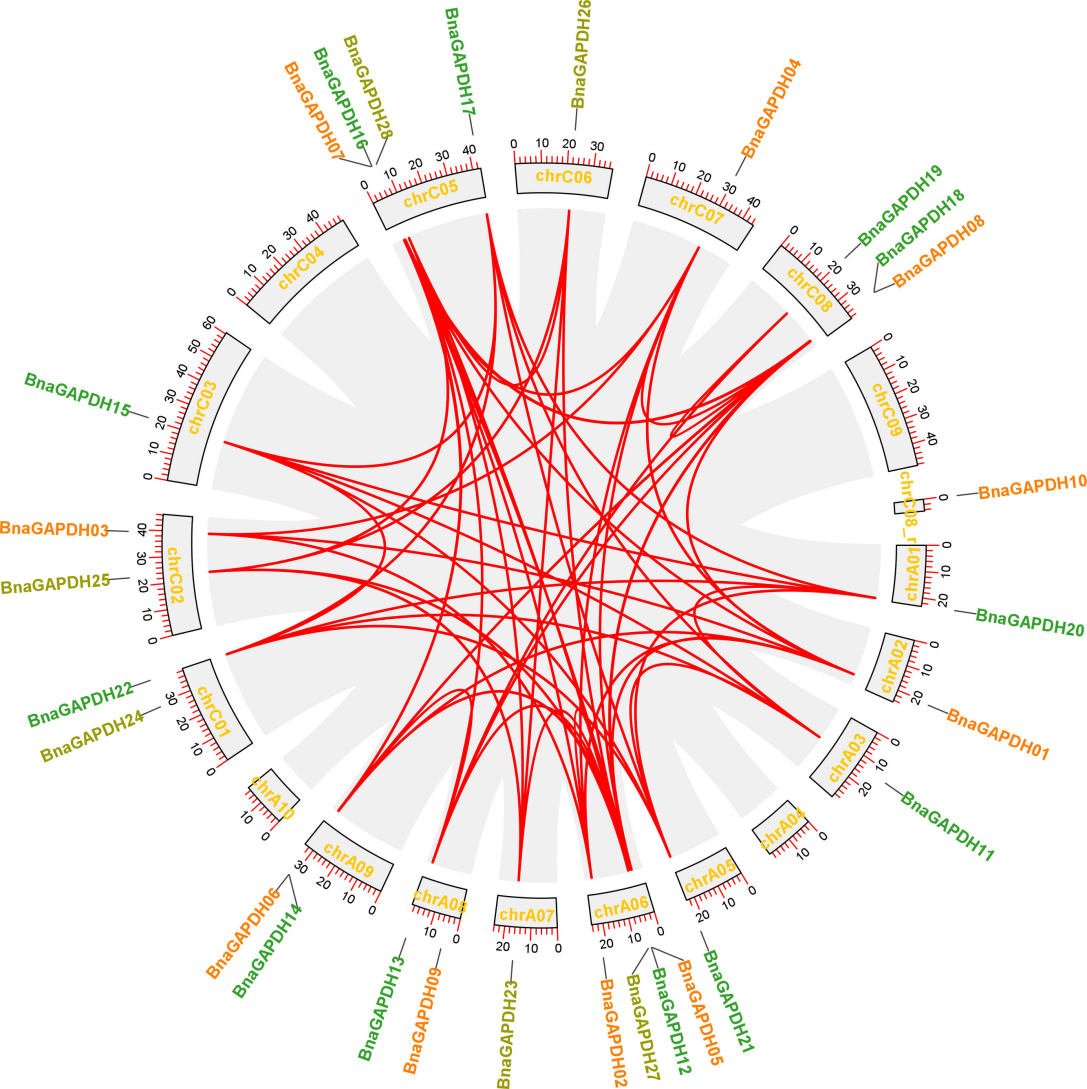
Schematic representations for the chromosomal distribution and inter-chromosomal associations of *B.napus GAPDH* genes. Grey lines indicate all syntenic blocks in the *B. napus* genome, and the red lines indicate syntenic *BnaGAPDH* gene pairs. The chromosome numbers are indicated at the bottom of each chromosome.

A chromosomal locational analysis was performed to gain insights into the distribution of *GAPDH* family genes on the chromosomes of *B. napus*. A total of 28 *BnaGAPDHs* were distributed unevenly on 16 chromosomes of *B. napus*, 13 genes in the An-subgenome, and 15 genes in the Cn-subgenome (**Figure 2**). ChrA06 and ChrC05 contained four *GAPDH* genes, whereas ChrA01, ChrA02, ChrA03, ChrA07, ChrC03, ChrC06, and ChrC07 had only one. Furthermore, potential gene duplication events in the *B. napus* genome were analyzed (**Figure 2** and **Supplementary Table S2**). 58 whole-genome duplication (WGD)/segmental duplication events with 25 *BnaGAPDHs* were detected in the *B. napus* genome, indicating that WGD and segmental duplication were important in the expansion of the *B. napus GAPDH* gene family.

**Figure 3:**
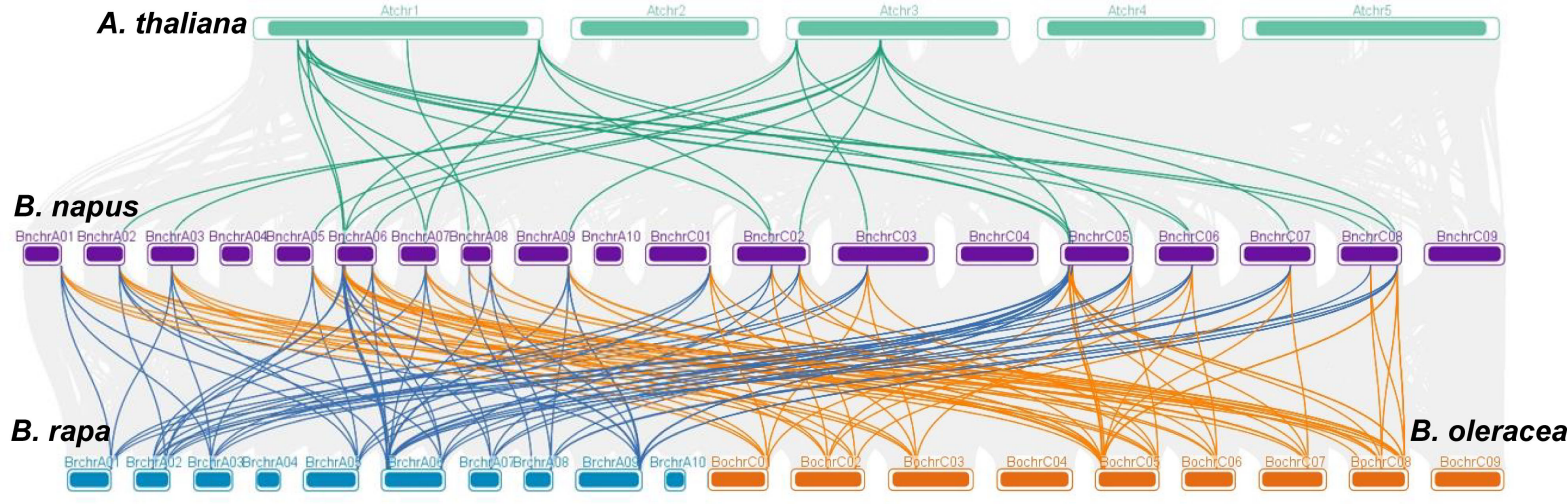
Syntenic relationships of *GAPDH* genes between *B. napus*, *B*. *rapa*, *B*. *oleracea*, and *A. thaliana*. The four species of plants chromosomes are shown in different colors. Grey lines in the background show the collinear blocks between *B.napus* and other 3 plant genomes, the colored lines highlight the syntenic *GAPDH* gene pairs.

Collinearity analysis was conducted involving *B. napus*, *B. rapa*, *B. oleracea*, and *A. thaliana* to further explore the evolutionary mechanisms of the *BnaGAPDH* genes (**Figure 3**; **Supplementary Table S3**). A total of 23 *BnaGAPDHs* had a collinear relationship with 6 At*GAPDH*s in *A. thaliana*, whereas 26 *BnaGAPDHs* were collinear with 14 *BraGAPDH*s in *B. rapa* and 14 *BolGAPDH*s in *B. oleracea*. Notably, most of the orthologs of *A. thaliana* (6/7, 85.7%), *B. rapa* (14/14, 100%), and *B. oleracea* (14/14, 100%) sustained a syntenic association with *BnaGAPDHs*, suggesting that WGD played a key role in *BnaGAPDH* gene family evolution along with segmental duplication. Also, nonsynonymous and synonymous substitution ratio (Ka and Ks) analyses of orthologous *GAPDH* gene pairs were performed to detect the driving force for the evolution of the *GAPDH* gene family (**Supplementary Table S3**). The results showed that most of the orthologous *GAPDH* gene pairs had a Ka/Ks ratio of less than 1, suggesting purifying selective pressure during *GAPDH* gene family evolution and conserved functions of these genes. Only one gene pair, *BnaGAPDH26* (*BnaC06g19790D*) and *BolC6t38145H*, had a Ka/Ks ratio of more than 1, indicating that these genes had undergone positive selection pressure and might have evolved new functions to help plants cope with their living environments.

### 3.4 Conserved motifs, structural domains, and gene structure analyses of *BnaGAPDHs* in *B. napus*

**Figure 4:**
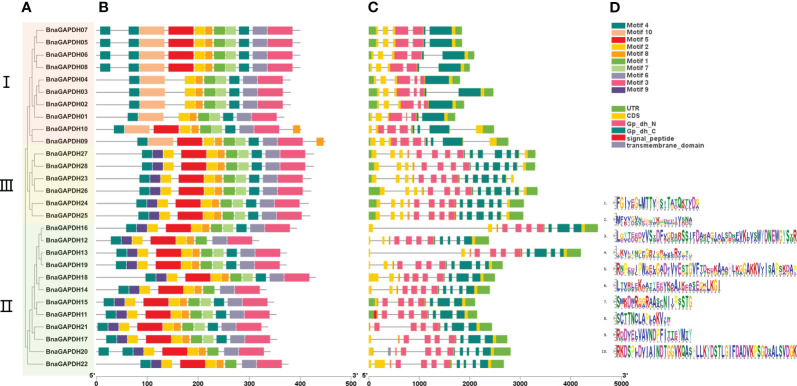
The motif analysis and gene structure of the *GAPDH* family genes from *B.napus*. **(A)** Phylogenetic tree of 28 *BnaGAPDHs*. According to the phylogenetic relationships, the *GAPDH* genes from *B.napus* genome were clustered into three groups (Groups I–III). **(B)** Conserved motifs identified in the BnGAPDHs. Different color boxes show motifs 1 to 10. **(C)** The overlying of gene structure and conserved domains of the BnGAPDHs. Light green color shows the UTR regions, yellow color shows the CDS or exons (pink color shows the specific Gp_dh_N domain (PF00044), dark green color shows Gp_dh_C domain (PF02800), red color shows transmembrane domain, and grey color shows signal peptide), black horizontal line shows the introns. **(D)** The corresponding sequence logos of 10 conserved motifs.

Ten motifs and the association with conserved domains were predicted in the BnaGAPDH proteins (**Figure 4B** and **Supplementary Table S4**). Motif 5 and Motif 10 belonged to the Gp_dh_N domain, whereas Motifs 1, 3, 4, 6, 7, and 8 belonged to the Gp_dh_C domain. Most members from the same group shared similar motif compositions, whereas the motif compositions varied slightly among groups (**Figures 4A, B**). Motifs 1, 2, 3, 4, 6, 7, and 8 were identified in all *BnaGAPDHs* except for four genes (*BnaGAPDH12*, *BnaGAPDH14*, *BnaGAPDH20*, and *BnaGAPDH22*). Motif 10 was found only in Group I, whereas Motif 9 was present only in Groups II and III. Taken together, these results indicated that the motif compositions within the group were relatively consistent, providing additional support for the phylogenetic classification.

Subsequently, the gene structure of the 28 *BnaGAPDHs* were investigated (**Figure 4C**). The number of exons varied, ranging from 5 to 14. Most of the genes within the same cluster displayed similar exon-intron structures. Notably, members of Group III contained the highest number of exons, with each having 14, while those in Group I had the fewest. According to the protein structure predictions, all members possessed two domains specific to full-length GAPDHs: the Gp_dh_N domain and the Gp_dh_C domain. Interestingly, within Group III, BnaGAPDH11 and BnaGAPDH20 were unique, with the former appearing to have a signal peptide domain and the latter a transmembrane domain. These results demonstrated the presence of highly conserved structures within the subgroups and diversity among different groups. They indicated that the gaining and splitting of exons and introns, as well as the presence of highly conserved domains, were characteristic developments within the *BnaGAPDH* gene family.

### 3.5 *Cis*-acting element analysis of *BnaGAPDHs*

**Figure 5:**
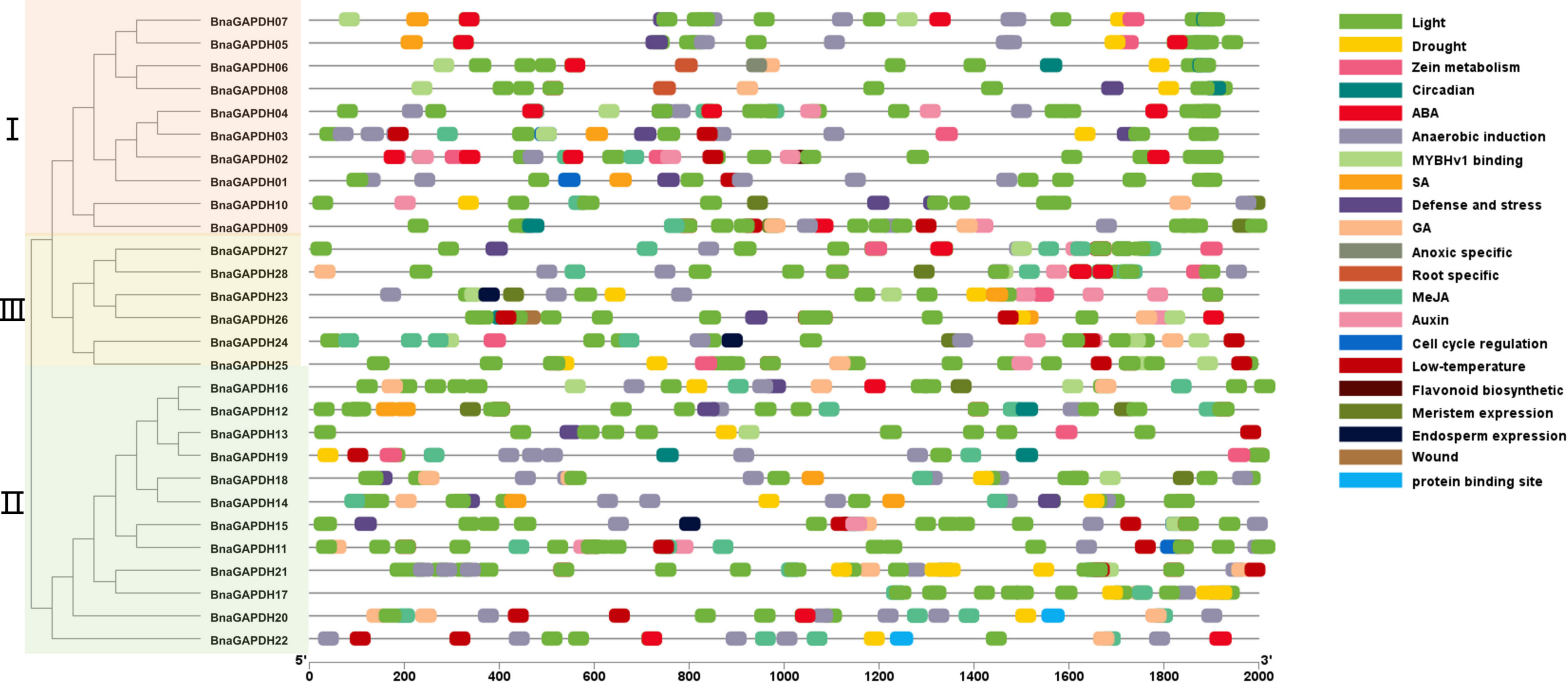
*Cis*-elements in the 2.0-kb upstream regions of the *BnGAPDH* family genes in *B. napus*. The cis-acting regulatory element analysis in the promoter region (2.0 kb upstream of translation initiation site) of *BnaGAPDH* genes was performed using the PlantCARE database. The gray horizontal line represents the promoter region with the length of 2000 bp. The forward 2.0 kb of the gene coding start site is used as the starting point of promoter analysis, marked as 5’0bp, and to the coding starting site as the end point of the promoter region, marked as 2000bp.Different color boxes show different identified cis-elements. The details of the cis-elements are provided in **Supplementary Table S5**. Different color boxes show different identified *cis*-elements.

The *cis*-elements in the 2-kb promoter regions were examined to recognize the gene functions and regulatory patterns of the *BnGAPDH* genes. A series of important *cis*-elements were identified (**Figure 5** and **Supplementary Table S5**), including abiotic, hormone-, defense-, and development-responsive elements. Defense- and stress-related elements (TC-rich repeats) were predicted in the promoter regions of 13 *BnaGAPDH* genes, and 12 of these 13 genes additionally contained 1–3 hormone-responsive elements related to ABA (ABRE), SA (TCA-element), gibberellin (GARE-motif/P-box), and MeJA (TGACG/CGTCA-motif). Other *cis*-elements, such as drought-, wound-, low-temperature-, light-, and anaerobic-responsive elements, were also present in the promoter regions of *BnGAPDH* genes. These findings suggested that the expression of the *BnGAPDH* genes was regulated by various environmental factors.

### 3.6 Expression patterns of *BnaGAPDH* genes in multiple tissues

**Figure 6:**
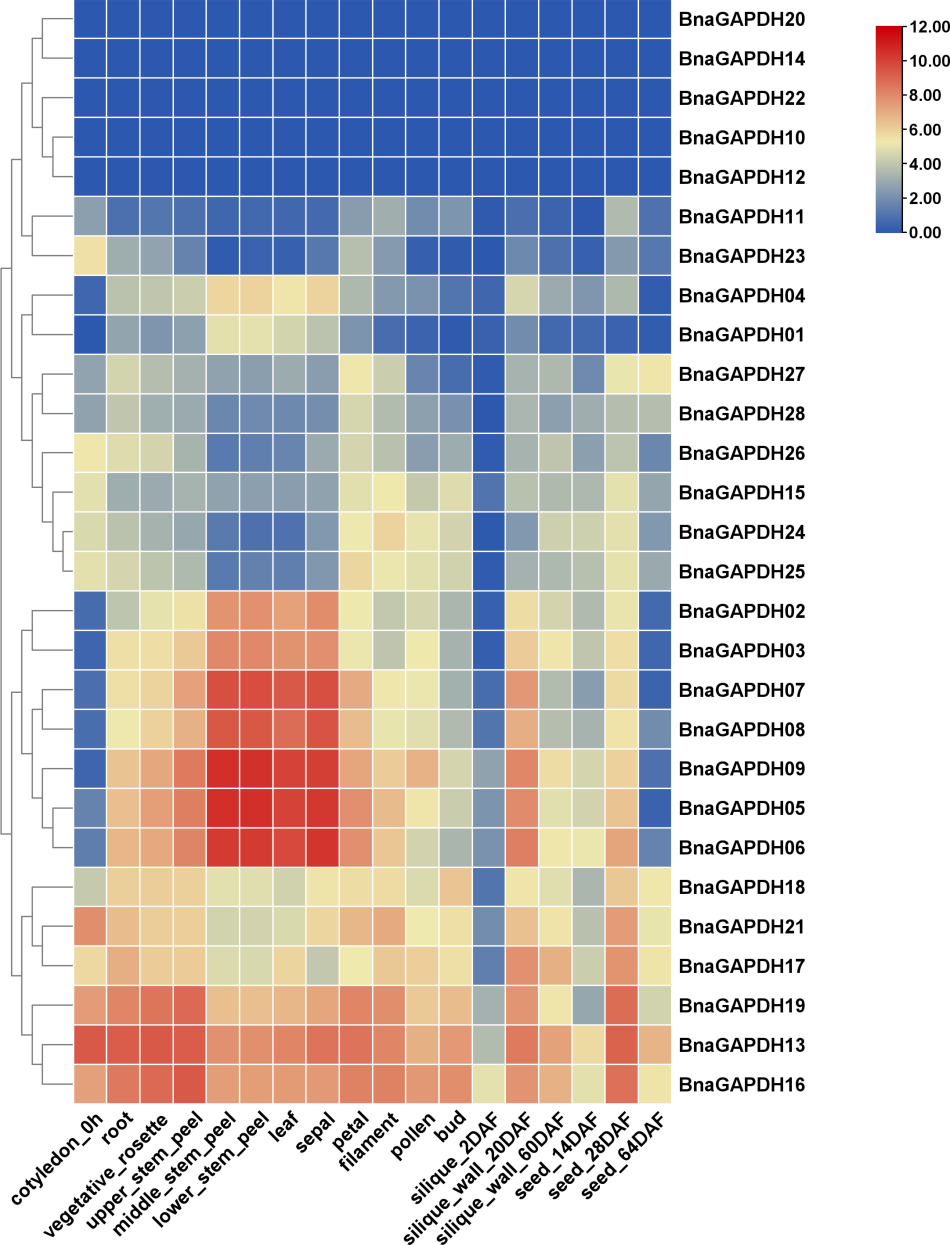
Expression patterns of *BnGAPDH* family genes in different tissues. The expression data were gained from the RNA-seq data and calculated by fragments per kilobase of exon model per million (FPKM) values. The label below the heatmap represents the different tissues of *B. napus* ZS11, the right side of the heatmap represents different *BnGAPDH* genes. The color bar represents log2 (FPKM+1) values. All values were detailed in **Supplementary Table S6**.

The expression patterns of *BnaGAPDHs* were investigated in the cotyledons, roots, rosettes, stems, leaves, sepals, filaments, pollens, buds, siliques, silique walls, and seeds in different developing stages to assess the functional properties of *GAPDH* genes in *B. napus* (**Figure 6** and **Supplementary Table S6**). The results showed that 3 genes were expressed (FPKM > 1) in at least 1 stage, 12 genes showed high expression (FPKM > 100), and 11 genes were lowly expressed (1 < FPKM < 100). All Group I *BnaGAPDHs* except for *BnaGAPDH10* were highly expressed in stems, leaves, and sepals, but lowly expressed in cytoledon_0h, silique_20 DAF, and seed_64DAF. Six Group II genes (*BnaGAPDH13*, *BnaGAPDH16*, *BnaGAPDH17*, *BnaGAPDH18*, *BnaGAPDH19*, and *BnaGAPDH21*) were consistently expressed at high levels in most tissues, whereas Group III members exhibited relatively low expression levels in most tissues except the silique_20 DAF stage. The other five *BnaGAPDH* genes showed weak or no expression in different tissues. The diverse expression patterns among groups indicated that *BnaGAPDH* genes might exert different functions in growth and development.

### 3.7 Expression patterns of *BnGAPDH* genes in response to *S. sclerotiorum* infection

**Figure 7:**
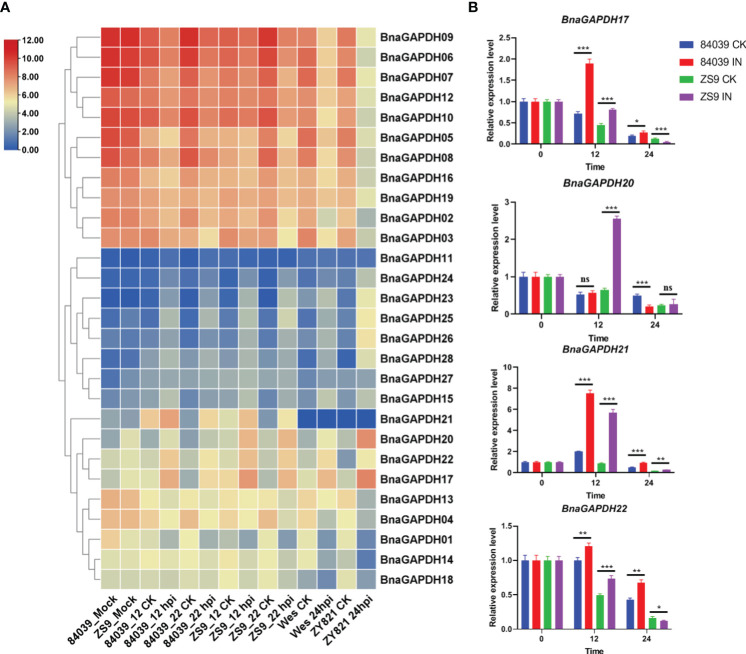
Expression patterns of *BnaGAPDH* genes in different cultivar of *B. napus* inoculated with *S. sclerotiorum*. **(A)** Expression patterns of *BnaGAPDH* genes in *B. napus* cultivars 84039, ZS9, Westar and ZY821 inoculated with *S. sclerotiorum*. Expression data were gained from the RNA-seq data and calculated by fragments per kilobase of exon model per million (FPKM) values. The expression levels of each gene were normalized by log2 (FPKM+1) in each sample, as indicated by different color rectangles. All values were detailed in **Supplementary Table S7**. **(B)** RT-qPCR analysis of *BnGAPDH17*, *BnGAPDH20*, *BnGAPDH21* and *BnGAPDH22* in *B. napus* cultivars 84039 and ZS9 inoculated with *S. sclerotiorum*. Data were calculated by the method of 2^-ΔΔCT^. * p<0.05, **p<0.01, ***p<0.001.

The expression profiles of 28 *BnaGAPDHs* in four *B. napus* cultivars (sensitive cultivar 84039 and Westar and moderately resistant cultivar ZS9 and ZY821) inoculated with *S. sclerotiorum* were investigated to assess the role of *BnaGAPDH* genes in response to biotic stress responses (**Figure 7A**, **Supplementary Table S7**). *BnaGAPDH02*, *BnaGAPDH03*, *BnaGAPDH05*, *BnaGAPDH06*, *BnaGAPDH07*, *BnaGAPDH08*, *BnaGAPDH09*, *BnaGAPDH10*, *BnaGAPDH12*, *BnaGAPDH16*, and *BnaGAPDH19* were highly expressed at all time points in four cultivars, but downregulated in the four cultivars after *S. sclerotiorum* infection compared with the control. Moreover, *BnaGAPDH17*, *BnaGAPDH20*, and *BnaGAPDH22* were highly upregulated in the four cultivars after *S. sclerotiorum* infection, whereas *BnaGAPDH01*, *BnaGAPDH04*, *BnaGAPDH13*, *BnaGAPDH14*, and *BnaGAPDH18* were downregulated, indicating that these genes might positively/negatively facilitate resistance against *S. sclerotiorum* in *B. napus*. While *BnaGAPDH21* was not detected in Westar and ZY821, it was highly upregulated in cultivar 84039 and ZS9 during *S. sclerotiorum* infection. Other *BnaGAPDHs* showed lower expression in all samples. Taken together, *BnaGAPDH* genes exhibited variable expression patterns in response to necrotrophic biotic stress.

To verify the aforementioned results, RT-qPCR was performed focusing on the expression levels of *BnaGAPDH17*, *BnaGAPDH20*, *BnaGAPDH21*, and *BnaGAPDH22* in cultivars ZS9 and 84039 during *S. sclerotiorum* inoculation (**Figure 7B**). Our results closely mirror those obtained from the overall transcriptome sequencing, demonstrating varying degrees of upregulation for all four genes during *S. sclerotiorum* infection. In the moderately resistant cultivar ZS9, the four genes exhibited a consistent pattern of upregulation at the early stage (12 hpi), followed by downregulation at the later stage (24 hpi) in response to *S. sclerotiorum* infection. However, notable discrepancies were observed in the susceptible cultivar 84039. Specifically, both *BnaGAPDH17* and *BnaGAPDH22* showed significant upregulation at both 12 and 24 hpi in 84039, while *BnaGAPDH20* displayed no significant upregulation and even underwent downregulation in the later stages of infection. Interestingly, the sole gene that exhibited consistent upregulation in both cultivars was *BnaGAPDH21*. These results imply that the response of these genes to *S. sclerotiorum* infection varies between cultivars, suggesting potential disparities in the underlying mechanisms of resistance to the pathogen between ZS9 and 84039.

### 3.8 Expression patterns of *BnGAPDH* genes under hormonal and OA treatments

**Figure 8:**
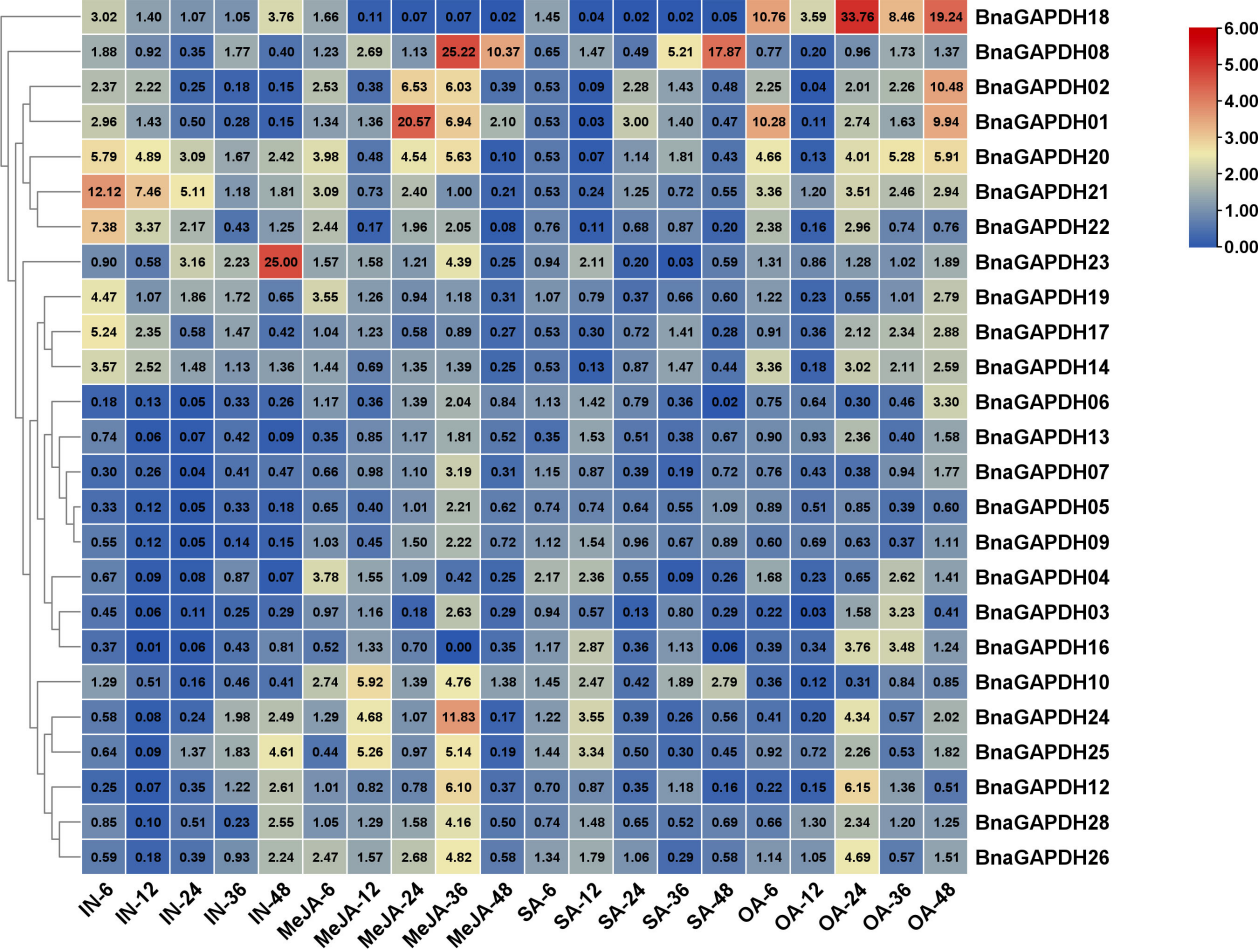
RT-qPCR analysis of *BnGAPDH* genes under plant hormones treatments. Expression profiles of *BnGAPDH* genes in leaves of *B. napus* cultivar ZS9 during inoculation of *S. sclerotiorum* and treatment with MeJA, SA, and OA. Blue and red colors are used to represent low-to-high expression levels, and colors scales correspond to the fold-change values compared with the counterpart control. RT-qPCR data were calculated by the method of 2^-ΔΔCT^.

Phytohormones SA, MeJA, and OA, as key factors of plant–*S. sclerotinia* interaction, were used to treat *B. napus* seedlings to further investigate the possible involvement of *BnaGAPDHs* in signaling pathways during *B. napus* – *S. sclerotirum* ineraction (**Figure 8**). The relative expression levels of *BnaGAPDHs* detected by qRT-PCR showed that *BnaGAPDHs* were differentially affected by SA, MeJA, and OA with significant changes. After inoculation with *S. sclerotiorum*, the relative expression of *BnaGAPDH01*, *BnaGAPDH02*, *BnaGAPDH14*, *BnaGAPDH17*, *BnaGAPDH18, BnaGAPDH19*, *BnaGAPDH20*, *BnaGAPDH21* and *BnaGAPDH23* was slightly upregulated in the early stage of MeJA treatment and the late stage of OA treatment, but they were almost not induced by SA (**Figure 8**). *BnaGAPDH18* was upregulated in the early stage (6 hpi) and late stage (48 hpi) of *S. sclerotiorum* infection. After OA treatment, the expression level showed an upward trend, but it was almost not affected by MeJA and SA. It was speculated that *BnaGAPDH18* might participate in the resistance of *S. sclerotiorum* through an MeJA/SA-independent signaling pathway. On the contrary, *BnaGAPDH08* was upregulated by MeJA and SA, whereas *S. sclerotiorum* infection and OA treatment had no significant effect on its expression. Taken together, the response of *BnaGAPDHs* to jasmonic acid (JA) and OA was consistent with that of *S. sclerotiorum*, but it tended to be independent of the SA signaling pathway.

### 3.9 Subcellular localizations of BnaGAPDH17, BnaGAPDH20, BnaGAPDH21, and BnaGAPDH22

**Figure 9:**
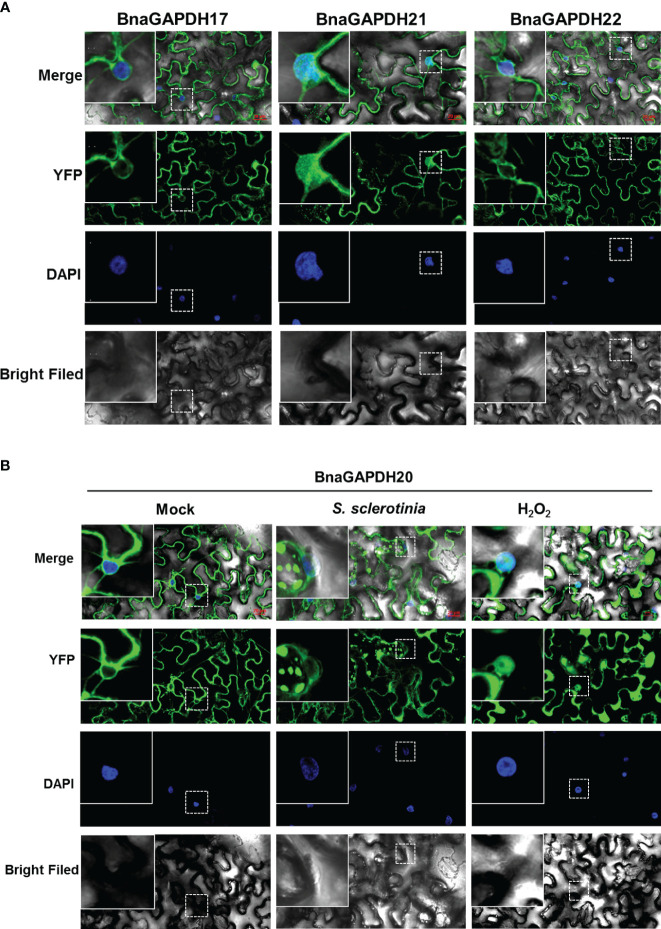
Subcellular localization of BnaGAPDH17, BnaGAPDH20, BnaGAPDH21 and BnaGAPDH22. **(A)** Subcellular localization of BnaGAPDH17, BnaGAPDH21 and BnaGAPDH22 under normal condition. **(B)** Subcellular localization of BnaGAPDH21 under normal condition (mock), *S. sclerotiorum* infection and H_2_O_2_ treatment. The nuclei were stained by 4’,6-diamidino-2-phenylindole (DAPI).

The aforementioned expression analysis revealed that four *BnaGAPDHs* (*BnaGAPDH17*, *BnaGAPDH20*, *BnaGAPDH21*, and *BnaGAPDH22*) exhibited a more significant up-regulation trend than other *BnaGAPDHs* in response to various stimuli, including cold stress, *S. sclerotiorum* infection, OA treatment, and MeJA treatment. Consequently, it became necessary to investigate their biological functions. Clarifying their intracellular localization is a prerequisite for understanding these functions. The transient expression assay in tobacco leaves revealed that BnaGAPDH17, BnaGAPDH20, and BnaGAPDH22 had similar localizations, primarily in the cytoplasm, and were also distributed near the nucleus and the plasma membrane (**Figures 9A, B**). However, they were not present in the inner nucleus. The aforementioned subcellular localization was consistent with the predicted results. In contrast, BnaGAPDH21not only clustered near the nucleus and plasma membrane but obviously clustered inside the nucleus (**Figure 9A**).

GAPC-types of *A. thaliana* are reported to be localized in different subcellular structures depending on their oxidation state. *S. sclerotiorum* infection often causes the production of oxidizing substances, such as oxygen anion and hydrogen peroxide (Schneider et al., 2018). Hence, it was speculated that *S. sclerotiorum* infection might cause the nuclear transfer of GAPC-type BnaGAPDHs. YFP-*BnaGAPDHs* were transiently expressed in tobacco, followed by the inoculation of *S. sclerotiorum* to test this hypothesis. As a positive control, tobacco leaves were treated with H_2_O_2_. Similar to AtGAPCs, confocal microscopy observation revealed that BnaGAPDH20 treated with H_2_O_2_ significantly aggregated in the nucleus (**Figure 9B**). *YFP-BnaGAPDH20* was found to aggregate in the nucleus of individual cells at the disease–health junction of tobacco leaves infected by *S. sclerotiorum* (**Figure 9B**). However, the *S. sclerotiorum* infection induced nuclear translocalization was less than that of H_2_O_2_ treatment. Still, many cells were not aggregated in the nucleus even infected by *S. sclerotiorum*, which might be related to the complex interaction mechanism between *S. sclerotiorum* and plants.

## 4 Discussion

Plants have intricate and sophisticated signaling networks that are ubiquitous, similar to those observed in animals. These networks elicit diverse cellular responses to various input signals, including growth promotion and PCD. Plants require efficient regulatory systems that integrate environmental and developmental signals across various tissues to achieve equilibrium. The multifunctional central hub, glyceraldehyde-3-phosphate dehydrogenase (GAPC), plays a crucial role in detecting and counterbalancing metabolic and redox imbalances. Moreover, GAPDH facilitates various modifications of sensitive cysteine residues in a finely-tuned manner, responding to external stressors of different degrees and types (Schneider et al., 2018; Lazarev et al., 2020; Sirover, 2021). Compared with animals, plants harbor more GAPDH isoforms, potentially collaborating to facilitate precise signal transmission and response, thereby restoring and maintaining a dynamic balance (Henry et al., 2015; Xie et al., 2021). Ultimately, PCD is initiated as a last resort when metabolism spins out of control, serving to mitigate damage to surrounding cells.

Numerous studies have indicated that various subtypes of GAPDHs in *Arabidopsis* occupy specific subcellular locations depending on their oxidation state (Schneider et al., 2018). When the cellular redox state shifts toward oxidation under stress, a portion of the redox-sensitive GAPDH undergoes oxidation, prompting its relocation to the nucleus, potentially triggering changes in gene expression (Schneider et al., 2018). For example, GAPDH has been observed in the nuclei of root cells exposed to cadmium (Vescovi et al., 2013), as well as in calcium-stressed and unstressed tobacco BY-2 cells (Testard et al., 2016). The “moonlighting” functions of GAPDH have been elucidated in an increasing number of studies (Tristan et al., 2011; Nicholls et al., 2012; Sirover, 2012; Hildebrandt et al., 2015). In this study, the nuclear location of BnaGAPDH21 was observed in certain cells during *S. sclerotiorum* infection but not in others. *S. sclerotiorum* is an aggressive pathogenic fungus marked by a short biotrophic phase during the infection process. Both the fungus and hosts employ a complex array of regulatory mechanisms to generate and accumulate ROS for survival, with the plant encouraging ROS production to stave off the pathogen, but biotrophic or hemi-biotrophic pathogens tend to scavenge ROS for their own survival. However, the influence of *S. sclerotiorum* on ROS during early infection is obscure and may implicate intricate regulatory mechanisms. The precise transition point of *S. sclerotiorum* from biotrophic to necrotrophic growth is not well-defined. Consequently, in cells at the forefront of the plant –*S. sclerotiorum* interaction, the redox state might fluctuate significantly. Moreover, other complex biological processes, including signal transduction, gene expression regulation, and additional factors, may also contribute to the nuclear translocation of BnaGAPDH20 during plant–pathogen interactions. Thus, a multi-faceted exploration of the mechanisms governing plant–pathogen interactions is essential for a more comprehensive understanding of the reasons behind the nuclear translocation of BnaGAPDH20.

As a necrotrophic pathogen, *S. sclerotiorum* secretes cell wall–degrading enzymes and toxins such as OA to establish infestation (Magro et al., 1984; Heller and Tudzynski, 2011). Conversely, plants have developed a range of defense mechanisms involving signaling networks, enabling them to survive and maintain their fitness (Zeng et al., 2018). These defense responses commonly begin with the recognition of pathogen-associated molecular patterns by plant pattern recognition receptors, subsequently activating pattern-triggered immunity (PTI). Alternatively, the detection of effectors by plant resistance (R) gene products triggers effector-triggered immunity (Jones and Dangl, 2006; Henry et al., 2013). To date, the immune response mechanisms of *B. napus* to *S. sclerotiorum* have been limited to PTI, although several effectors have been identified (Xiao et al., 2014; Yang et al., 2018; Wang et al., 2019; Tang et al., 2020).

The induced resistance response in plants is regulated by a complex signaling network and involves the expression of a series of resistance-related genes. Specifically, in the *B. napus*−*S. sclerotiorum* pathosystem, *B. napus* employs a variety of signaling pathways, involving the mitogen-activated protein kinase cascade reaction, ROS, SA, and JA, to activate resistance to *S. sclerotiorum* (Ding et al., 2021). Although some of the plant–*S. sclerotiorum* interaction mechanisms are now determined, these perceptions are still just the tip of the iceberg. There are several researches focusing on *GAPDH*’s functions of regulating gene expression including function as a translational suppressor of AT1R and mediates the effect of H_2_O_2_ on AT1R mRNA (Backlund et al., 2009), regulating cyclooxygenase-2 and endothelin-1expression by targeting mRNA stability possibly by a novel, redox-sensitive mechanism (Rodriguez-Pascual et al., 2008; Ikeda et al., 2012). The mechanism by which GAPDH regulates gene expression is not yet fully understood, but research has found that GAPDH acetylation may be required for GAPDH dependent apoptotic gene regulation involving p53, PUMA, Bax and p21 (Sirover, 2021). In this study, the association of GAPDHs with ROS, SA, and MeJA, as well as with *S. sclerotiorum*, strongly suggested an potential redox-sensitive mechanism of GAPDHs participate in the immune response of *B. napus* against *S. sclerotiorum.*

In this study, 12 out of 13 *BnaGAPDH* genes containing defense and stress response elements were also found to contain 1–3 types of hormone-responsive elements, indicating their diverse roles in various stress regulatory networks. Many studies have shown the involvement of GAPDHs with abiotic stresses besides their roles in the immune responses. In *Arabidopsis*, AtGAPDHs could interact with E3 ubiquitin ligase (SINAL7), which positively controlled drought resistance and delayed leaf senescence of *Arabidopsis* plants, revealing the involvement of GAPC in stress tolerance (Peralta et al., 2018). Furthermore, GAPDHs may play important roles in response to drought stress by interacting with PLDδ or pLDα1 to promote ABA-regulated stomata closure (Hong et al., 2008; Zhang et al., 2020). Therefore, the identification of BnaGAPDHs interacting proteins is of great significance for further understanding their functional mechanisms in plant resistance to *S. sclerotiorum*.

## Data availability statement

The datasets presented in this study can be found in online repositories. The names of the repository/repositories and accession number(s) can be found in the article/**Supplementary Material**.

## Author contributions

JX: Conceptualization, Data curation, Formal analysis, Investigation, Methodology, Project administration, Software, Validation, Visualization, Writing – original draft, Writing – review & editing. RW: Data curation, Resources, Visualization, Writing – review & editing. XZ: Conceptualization, Investigation, Resources, Writing – review & editing. WZ: Data curation, Formal analysis, Investigation, Writing – original draft. YZ: Data curation, Investigation, Visualization, Writing – original draft. JL: Funding acquisition, Methodology, Project administration, Writing – review & editing. PZ: Conceptualization, Validation, Writing – review & editing. SC: Formal analysis, Funding acquisition, Software, Supervision, Writing – review & editing. HL: Conceptualization, Resources, Writing – original draft. AW: Conceptualization, Project administration, Supervision, Validation, Writing – review & editing. LC: Funding acquisition, Project administration, Supervision, Writing – review & editing.
