# Supplementary figures and images for: Identification and expression profiling of GAPDH family genes involved in response to Sclerotinia sclerotiorum infection and phytohormones in Brassica napus
Source: Front Plant Sci. 2024 Apr 30;15:1360024. doi: 10.3389/fpls.2024.1360024 (PMC11091349; doi:10.3389/fpls.2024.1360024)

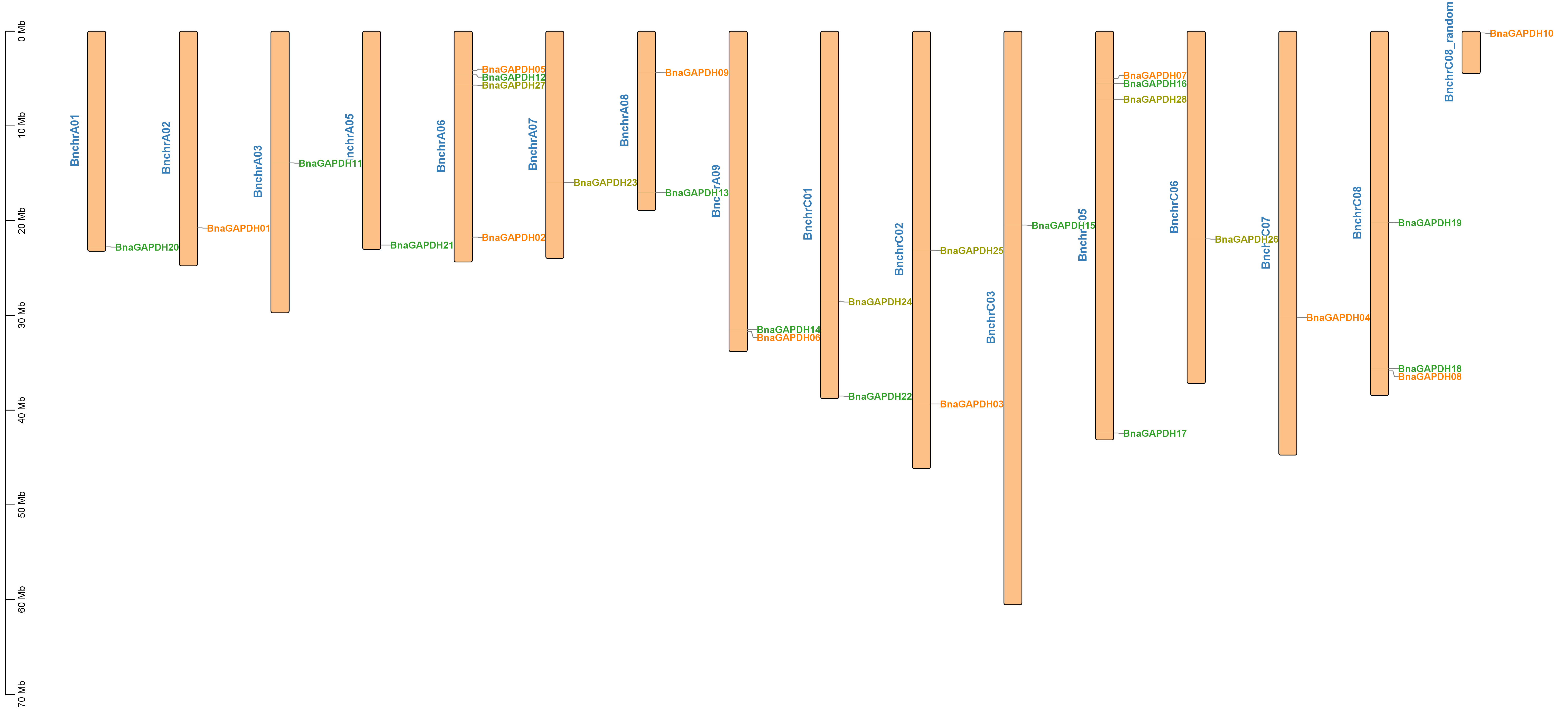

Supplement: Supplementary file 1 [file Image_1.tiff]
